# Enhancing Across-Population Genomic Prediction for Maize Hybrids

**DOI:** 10.3390/plants13213105

**Published:** 2024-11-04

**Authors:** Guangning Yu, Furong Li, Xin Wang, Yuxiang Zhang, Kai Zhou, Wenyan Yang, Xiusheng Guan, Xuecai Zhang, Chenwu Xu, Yang Xu

**Affiliations:** 1 Key Laboratory of Plant Functional Genomics of the Ministry of Education/Jiangsu Key Laboratory of Crop Genomics and Molecular Breeding, College of Agriculture, Yangzhou University, Yangzhou 225009, China; mx120200746@yzu.edu.cn (G.Y.); lfr953215657yy@163.com (F.L.); seuwangxin@163.com (X.W.); dx120220139@stu.yzu.edu.cn (Y.Z.); mx120200737@yzu.edu.cn (K.Z.); mx120210754@stu.yzu.edu.cn (W.Y.); mx120220781@stu.yzu.edu.cn (X.G.); cwxu@yzu.edu.cn (C.X.); 2 Zhongshan Biological Breeding Laboratory/Jiangsu Co-Innovation Center for Modern Production Technology of Grain Crops, Yangzhou University, Yangzhou 225009, China; 3 International Maize and Wheat Improvement Center (CIMMYT), Mexico City 06600, Mexico; xc.zhang@cgiar.org

**Keywords:** across population, genomic prediction, population structure, maize hybrids

## Abstract

In crop breeding, genomic selection (GS) serves as a powerful tool for predicting unknown phenotypes by using genome-wide markers, aimed at enhancing genetic gain for quantitative traits. However, in practical applications of GS, predictions are not always made within populations or for individuals that are genetically similar to the training population. Therefore, exploring possibilities and effective strategies for across-population prediction becomes an attractive avenue for applying GS technology in breeding practices. In this study, we used an existing maize population of 5820 hybrids as the training population to predict another population of 523 maize hybrids using the GBLUP and BayesB models. We evaluated the impact of optimizing the training population based on the genetic relationship between the training and breeding populations on the accuracy of across-population predictions. The results showed that the prediction accuracy improved to some extent with varying training population sizes. However, the optimal size of the training population differed for various traits. Additionally, we proposed a population structure-based across-population genomic prediction (PSAPGP) strategy, which integrates population structure as a fixed effect in the GS models. Principal component analysis, clustering, and Q-matrix analysis were used to assess the population structure. Notably, when the Q-matrix was used, the across-population prediction exhibited the best performance, with improvements ranging from 8 to 11% for ear weight, ear grain weight and plant height. This is a promising strategy for reducing phenotyping costs and enhancing maize hybrid breeding efficiency.

## 1. Introduction

Maize (*Zea mays*) is one of the most widely grown food crops in the world and plays an important strategic role in ensuring food security and the effective supply of agricultural products [1]. The primary objective of maize breeding is to develop hybrid varieties that exhibit high yield potential across diverse environmental conditions. Taking advantage of heterosis is an effective strategy for enhancing maize yield. Genomic selection (GS) breeding is a technique that utilizes genome-wide markers to predict unknown phenotypes [2], and it is integral to hybrid breeding practices [3,4]. By applying GS technology, breeders are able to estimate the breeding value or phenotypes of all potential crosses of known parents through phenotypic identification and model construction within the training population. This approach enables them to perform field evaluations on the predicted superior crosses, thereby reducing the corresponding breeding cycle and costs [5].

In the context of GS breeding, predictions are not confined to individuals within a population or to those that are genetically similar to the training population. The across-population prediction presents a cost-effective strategy to leverage genetic information from one population to predict individuals in another, genetically distinct population. While traditional GS relies on training and validating models within genetically similar populations for higher prediction accuracy, across-population predictions enable the utilization of existing datasets, thus minimizing the need for expansive, population-specific data collection. Therefore, investigating effective strategies and prediction accuracy for across-population predictions is essential for enhancing the application of GS technology in breeding practices. Nevertheless, the complexity of across-population prediction is higher than that of within-population prediction, as it involves not only the training population size and marker density but also the kinship between the training and breeding populations. A comprehensive understanding of these influencing factors and their interactions is vital for improving the accuracy of across-population predictions. The genetic relationships between training and breeding populations significantly impact prediction accuracy [6,7,8,9].

When the genetic diversity of the training population significantly differs from that of the breeding population, or when the data originate from two entirely distinct populations with low genetic structure correlation, the potential accuracy of the predicted phenotypic values may be limited. The influence of training population composition on prediction accuracy was explored using five double haploid (DH) populations, revealing that combining unrelated populations with opposite linkage phases to the breeding population may result in negative or reduced prediction accuracies [10]. Additionally, the differences in linkage disequilibrium (LD) among different populations, as well as specific allele effects and allele frequencies, could result in low accuracy in across-population predictions [11,12,13,14]. Several studies have demonstrated that the accuracy of across-population GS in animals is often low and sometimes even negative [15,16,17]. Research conducted on wheat populations revealed that genomic prediction across populations demonstrated low accuracy and indicated that it should not be adopted in small populations [18]. Overall, across-population genomic prediction remains a challenge in the field of GS.

To improve the accuracy of across-population genomic prediction, it is essential to examine the optimal size and composition of the training population, particularly when dealing with a large training population [19,20]. The optimal size of the training population is influenced by the heritability of its traits, the genetic relationship between training and breeding populations, and the population structure. Taking population structure and genetic relationships into account can substantially improve the predictive ability of GS models [21,22]. In studies involving rice and wheat populations, the performance of five optimization criteria for selecting training populations was evaluated, indicating that the optimal selection criterion varies depending on the trait architecture and population structure [23]. Using a diversity panel and ten bi-parental crosses within a half-diallel mating design, the across-population predictive abilities for 15 grapevine traits were assessed. The results demonstrated that optimizing the training set with the mean of determination coefficient (CDmean), the mean of prediction error variance (PEVmean), and the mean additive relationship could improve predictive ability for specific traits, thereby highlighting the potential of across-population genomic prediction in grapevine breeding [24].

Large amounts of highly unbalanced historical data are often available in crop breeding programs, particularly when considering data collected across different years and locations. This imbalance is a challenge for accurately training prediction models. During the processing of phenotypic data, researchers often implement strategies such as utilizing Best Linear Unbiased Prediction (BLUP) values to mitigate the phenotypic variations induced by differing environments and temporal factors, thereby reducing the influence of these variables. However, such operations cannot eliminate a series of complex factors such as epistatic effects between genes and the environment, heterosis, and parental inheritance. Therefore, incorporating known parental phenotypes, population structure, and environmental factors into the GS prediction model as covariates or fixed effects can help improve prediction accuracy and provide valuable insights across populations [25,26,27].

While previous research has conducted across-population predictions in crops such as wheat, rice, and maize, these studies have primarily focused on small populations [10,18,28]. To date, there is a lack of relevant investigations regarding across-population prediction within large maize hybrid populations, particularly when the phenotypic data are derived from BLUP values across various environments and locations. The goal of this study was to optimize across-population genomic prediction for maize hybrids. We proposed a population structure-based across-population genomic prediction (PSAPGP) strategy; this proposed PSAPGP enhances across-population genomic prediction for maize hybrids.

## 2. Results

### 2.1. Within- and Across-Population Prediction

**Figure 1:**
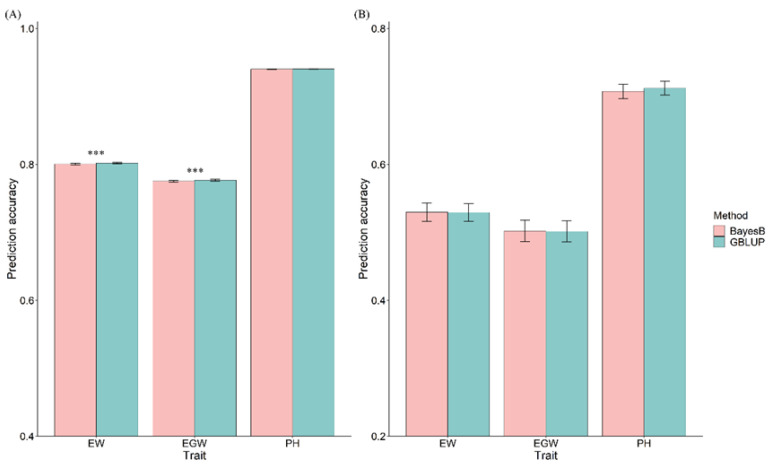
Prediction accuracy within the population under GBLUP and BayesB models. (**A**) Prediction accuracy of the training population; (**B**) prediction accuracy of the breeding population. Note: Asterisks indicate significant differences at *p* < 0.001 (***).

**Figure 2:**
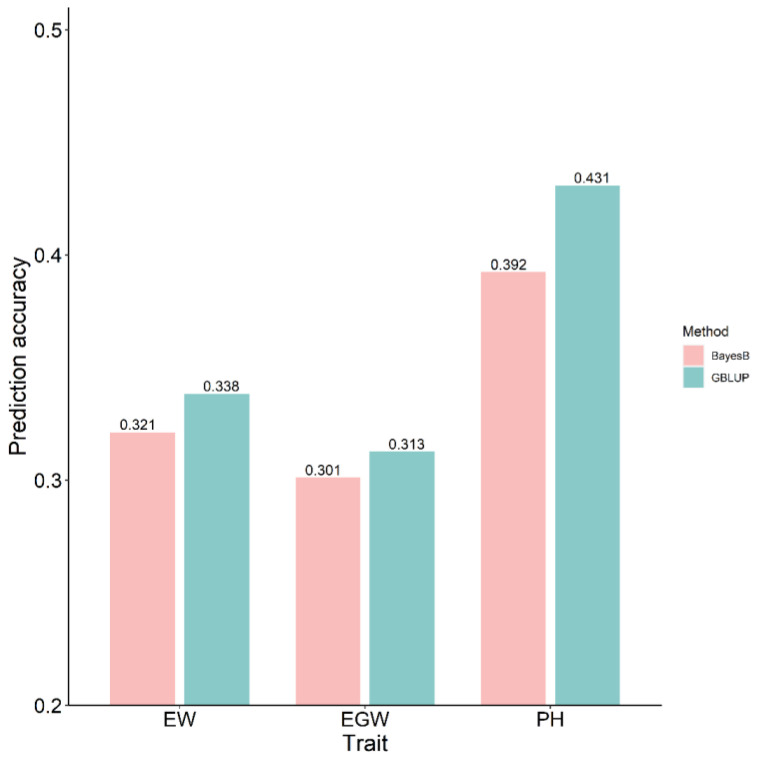
Across-population prediction accuracy using GBLUP and BayesB models.

The GBLUP and BayesB models were utilized to conduct genomic predictions both within and across populations, with prediction accuracy assessed using Pearson correlation coefficients between predicted and observed phenotypic values. Within the population, prediction accuracies varied between the training and breeding populations across the evaluated traits. Within the training population containing 5.820 maize hybrids (Pop1), the prediction accuracies for three traits, derived from 20 repeated five-fold cross-validations, ranged from 0.775 to 0.940 across various model–trait combinations. In the breeding population consisting of 523 maize hybrids (Pop2), prediction accuracies ranged from 0.502 to 0.712 for all model–trait combinations. Within the training population, the prediction accuracy for the EW and EGW traits showed significant differences between the two models, while PH did not exhibit significant differences. In the breeding population, the prediction accuracy for the three traits did not show significant variation among the different models (Figure 1). For the across-population genomic prediction, the phenotypic values in the breeding population were predicted based on the marker effects estimated from the training population. Figure 2 illustrates the accuracy of these across-population predictions. In the GBLUP model, the prediction accuracies for EW, EGW, and PH were 0.338, 0.313, and 0.431, respectively. In the BayesB model, the prediction accuracies for these traits were 0.321, 0.301, and 0.392, respectively.

### 2.2. Optimization of the Training Population

**Figure 3:**
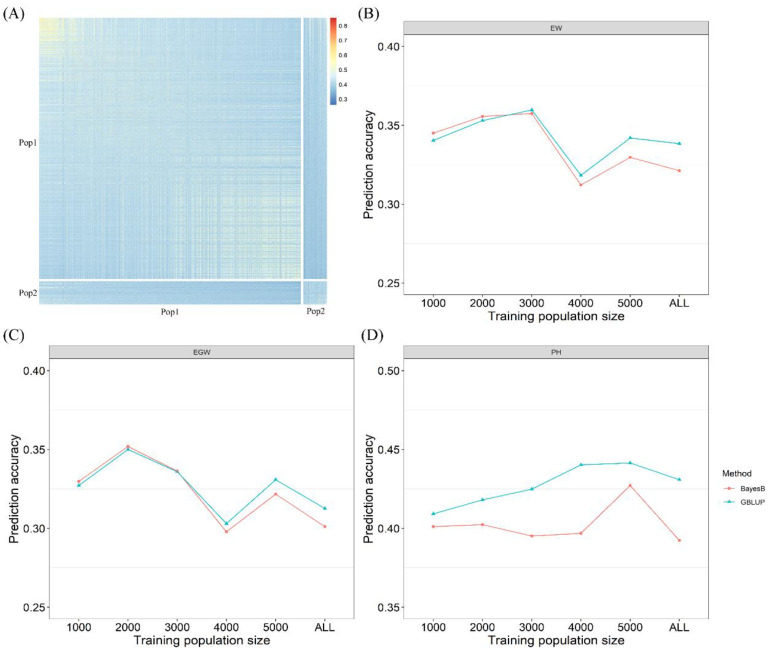
Across-population prediction accuracy of different training population sizes. (**A**) Heat map of kinship matrix for 6.343 maize hybrids; (**B**) prediction accuracy for ear weight; (**C**) prediction accuracy for ear grain weight; (**D**) prediction accuracy for plant height.

To enhance genomic prediction accuracy across populations, we optimized the training population based on the genetic relationships between the training and breeding populations. Initially, we constructed a genetic relationship matrix for 6.343 hybrids from Pop1 and Pop2. The genetic distance between each individual in Pop1 and Pop2 increased gradually from left to right (Figure 3A). Consequently, we selected five distinct training population sizes (1000, 2000, 3000, 4000, and 5000) based on the MeanRel and we assessed the prediction accuracy for these varying training population sizes. The results showed that for the traits EW and EGW, both the GBLUP and BayesB models improved the prediction accuracy when the training population size ranged from 1000 to 3000 (Figure 3B,C). Specifically, at a training population size of 2000, EGW exhibited the most obvious increase, with the GBLUP and BayesB models achieving enhancements of 12.0% and 16.9%, respectively. At a training population size of 3000, EW showed the highest increase, with the GBLUP and BayesB models yielding improvements of 6.3% and 11.3%, respectively. In contrast, for PH, the prediction accuracy exhibited the most significant increase when the training population size was between 4000 and 5000, peaking at a training population size of 5000, with the GBLUP and BayesB models achieving enhancements of 2.5% and 8.7%, respectively.

### 2.3. Population Structure-Based Across-Population Genomic Prediction

**Figure 4:**
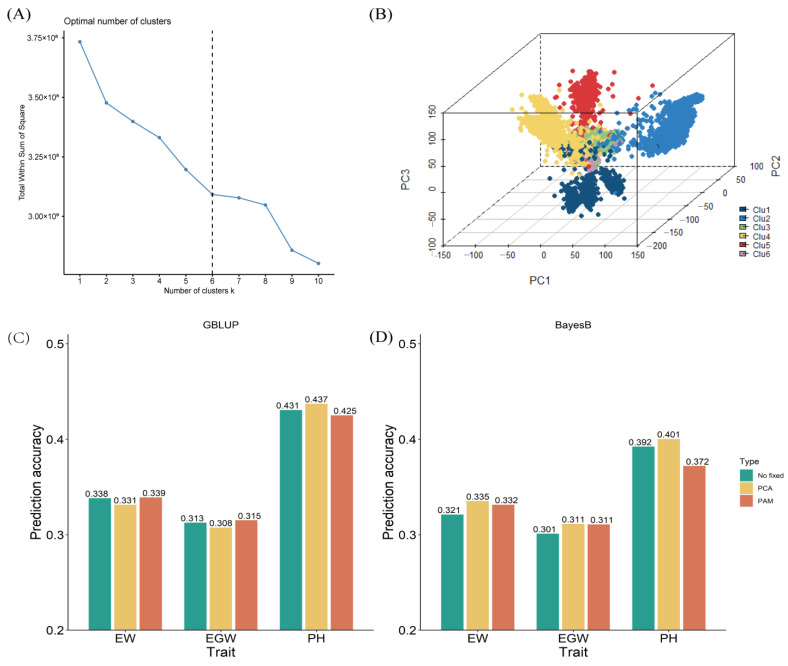
Population structure-based across-population genomic prediction using PCA and PAM as fixed effects; (**A**) scree plot to determine the optimal number of clusters; (**B**) three-dimensional PCA plot of the 6.343 maize hybrids; (**C**) across-population prediction accuracy with the GBLUP model; (**D**) across-population prediction accuracy with the BayesB model.

To investigate the effectiveness of the PSAPGP strategy, we employed PCA, PAM clustering, and Q-matrix analysis to assess the population structure of all hybrids from Pop1 and Pop2. Utilizing genotypic data comprising 58.876 SNPs from 6.343 hybrids, we determined that the optimal number of clusters was six (Figure 4A), after which we conducted the clustering analysis. A three-dimensional PCA plot was generated based on the clustering outcomes, clearly delineating the six categories (Figure 4B). The first six principal components derived from the PCA were subsequently integrated as fixed effects into the GBLUP and BayesB models. For the PAM results, the clustering results were transformed into six columns of data using One-Hot coding, which were also integrated into the GBLUP and BayesB models as fixed effects. The outcomes of the PSAPGP strategy, which integrated PCA and PAM into the GS models, are illustrated in Figure 4C,D. Notably, the integration of PCA and PAM clustering results into the GBLUP model did not yield a significant enhancement in across-population prediction accuracy; in fact, it resulted in a slight decrease. Conversely, when the BayesB model was utilized with the inclusion of PCA results, the prediction accuracy for EW, EGW, and PH exhibited increases of 4.4%, 3.4%, and 2.1%, respectively. In contrast, the integration of PAM results into the BayesB model led to increases in both EW and EGW traits by 3.2%, while PH experienced a minor decline.

**Figure 5:**
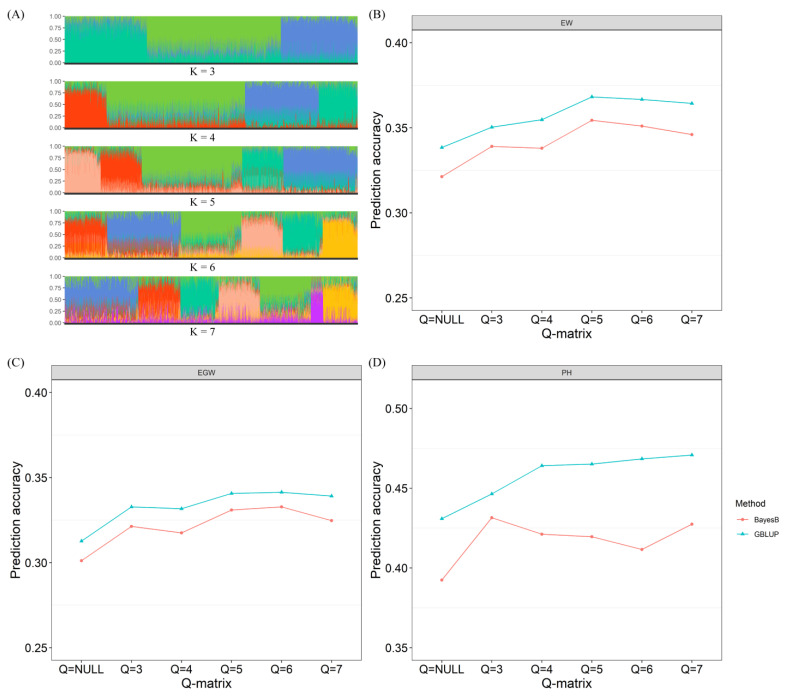
Population structure-based across-population genomic prediction using the Q-matrix as a fixed effect. (**A**) Population structure of the 6.343 maize hybrids at different K values; (**B**) across-population prediction accuracy for ear weight at different K values; (**C**) across-population prediction accuracy for ear grain weight at different K values; (**D**) across-population prediction accuracy for plant height at different K values.

The population structure Q-matrix of 6.343 hybrids was estimated using ADMIXTURE 1.3.0 software. We evaluated the population structure for K values of 3, 4, 5, 6, and 7 (Figure 5A), and calculated the prediction accuracy of different GS models when different Q-matrices were used as fixed effects. As illustrated in Figure 5, the prediction accuracy of the traits EW, EGW, and PH exhibited significant enhancements across all Q-matrix configurations. Notably, when K = 6 was integrated into the GS model, the prediction accuracies for EW, EGW, and PH improved by 8.4%, 9.2%, and 8.7%, respectively, within the GBLUP model. In the context of the BayesB model, the prediction accuracies for EW, EGW, and PH increased by 9.3%, 10.5%, and 4.9%, respectively. Utilizing the GBLUP method, the maximum observed improvement ratios for EW, EGW, and PH prediction accuracy across various Q-matrix configurations were 8.8%, 9.2%, and 9.3%, respectively. Conversely, under the BayesB method, the maximum improvement ratios for EW, EGW, and PH prediction accuracy across different Q-matrix configurations were 10.3%, 10.5%, and 10.0%, respectively (Figure 5B–D).

## 3. Discussion

In this study, we performed genomic predictions both within and across populations using the GBLUP and BayesB models. The lower accuracy of across-population predictions, as compared to within-population predictions, may be attributed to the great genetic distance between the training and breeding populations. When different populations exhibit large genetic distance, the estimation of marker effects can become inconsistent due to variations in alleles, allele frequencies, and linkage phases between the training and breeding populations [29]. Notably, the across-population prediction accuracy of GBLUP was found to be superior to that of BayesB without any optimization of the training population. However, when the training sizes were set at 1000 and 2000, the prediction accuracy of the BayesB method outperformed that of the GBLUP method for the EW and EGW traits. This finding suggests that the selection of the most effective method is contingent upon the genetic relationship between the training and breeding populations.

As breeders integrate GS into ongoing breeding programs, it is essential to carefully select the materials used to construct training populations and optimize the predictive abilities of models derived from these populations. To enhance the accuracy of across-population genomic prediction, previous efforts have focused on optimizing the training population. One study examined the effects of four different population selection methods in a winter wheat panel, finding that selection methods based on PEV outperformed both clustering and random methods [30]. Although employing the PEVmean algorithm to optimize the training population can be beneficial in certain contexts, particularly with small populations, the application of such optimization methods may impose considerable computational demands when larger populations are involved [31]. A method to optimize training sets using kinship, termed MeanRel, has been proposed. This method has demonstrated the potential in enhancing prediction accuracy in the across-population prediction of grapevine [24]. In the present study, we also employed this approach to optimize training populations based on kinship relationships. Our findings revealed varying degrees of improvement across different traits; however, we faced challenges due to the inconsistency in the number of optimal training populations selected for each trait. To address this issue and standardize the selection process, we proposed using the mean value of MeanRel as the cut-off criterion. This approach enabled us to identify the top 2.763 individuals from training populations that were most closely aligned with the breeding population. Under the GBLUP model, the prediction accuracy for the EW, EGW, and PH improved by 7.3%, 9.7%, and 0.07%, respectively. Similarly, under the BayesB model, the prediction accuracy for the same traits increased by 14.4%, 15.5%, and 1.9%, respectively. Notably, both EW and EGW are low-heritability traits. The results suggest that the strategy of optimizing training populations based on kinship has a more obvious advantageous for traits with low heritability.

Research has indicated that incorporating significant SNPs identified through Genome-Wide Association Study (GWAS) as fixed effects can improve the predictive ability of genomic prediction models [32,33]. Utilizing unbalanced phenotypic data collected across varying years or locations, along with markers associated with traits of interest as fixed effects, has been shown to significantly improve the prediction accuracy of key agronomic traits in wheat [30]. In the context of across-population genomic prediction, the accuracy can also be enhanced by integrating significant QTL into the model as fixed effects. The efficacy of across-population predictions is notably improved when the same QTL are present in two populations or when multiple genes are involved [18]. Nevertheless, the strategy of integrating population structure into across-population prediction models has not been extensively investigated. In this study, we propose the PSAPGP strategy to integrate the population structure into prediction models. To study the impact of different population structure evaluation methods on this strategy, we employed PCA, PAM clustering, and Q-matrix analysis to assess the population structure, using two GS models to demonstrate the proposed strategy. Under the GBLUP model, PCA and clustering did not yield significant effects. Conversely, the BayesB model exhibited a modest enhancement in performance. When the Q-matrix was integrated into the model as a fixed effect, both models demonstrated a substantial increase in the accuracy of across-population genomic prediction, with enhancements ranging from 8 to 11%. This improvement may be attributed to the fact that PCA and clustering analyses provide only a basic understanding of population genetic structure, whereas admixture population genetic structure analysis offers additional insights by predicting the optimal number of populations and exploring gene flow between them. Furthermore, it can assess the degree of admixture in individual samples, potentially providing more comprehensive information than the aforementioned methods, thereby positively influencing across-population predictions.

In the breeding of maize hybrids, the vast number of potential crosses makes extensive testing impractical. Identifying superior hybrids from this extensive pool using GS technology presents a long-term challenge. Utilizing public datasets of maize hybrids as a training population for across-population genomic prediction offers a highly cost-effective approach. If these accumulated data can be effectively applied, they will greatly improve the implementation and progress of maize GS breeding. Additionally, this approach of across-population genomic prediction leverages valuable genetic resources from diverse populations to enhance the genetic diversity of hybrids and expand the genetic foundation [28]. We propose a PSAPGP strategy that integrates population structure as a fixed effect in GS models to enhance the accuracy of genomic predictions across populations. Utilizing this strategy, we can further explore the implementation of non-additive effects, such as dominance and epistasis, in genomic prediction models to improve prediction accuracy across populations. Additionally, we may consider integrating multiple population datasets from various sources to expand the size of the training group. In the future, the application of machine learning and deep learning methods is anticipated to facilitate the development of an optimal model for across-population predictions, thereby increasing the accuracy of predictions across diverse populations [34,35]. To successfully implement the across-population GS breeding program, it is essential to establish an open-source GS breeding network along with shared molecular breeding platforms. This network should be capable of offering national agricultural research organizations and small- and medium-sized breeding companies access to advanced and comprehensive breeding technologies. These technologies include universal, efficient, and low-cost genotyping platforms such as target sequencing and liquid chip genotyping, standardized methods for collecting and organizing phenotypic data, and well-established modeling and prediction approaches [36,37,38]. By sharing breeding platforms and information, and integrating GS breeding strategies, we can continuously update the design and optimization models, improve the accuracy of across-population predictions for maize, and ultimately enhance the breeding efficiency of maize varieties.

## 4. Materials and Methods

### 4.1. Training Population of Maize Hybrids

This study utilized two distinct populations of maize hybrids. The first population, referred to as Pop1, serves as the training population, including 5.820 F1 hybrids derived from the cross of 194 maternal inbred lines which were a subset of the maize Complete-diallel plus Unbalanced Breeding-derived Inter-Cross (CUBIC) population and 30 diverse founder paternal lines. The 5.820 F1 hybrids were grown in five locations in 2015 for phenotypic data collection. The analysis focused on three traits: ear weight (EW), ear grain weight (EGW), and plant height (PH). To reduce the impact of environmental variability on the phenotypic data, BLUP values were computed, as referenced in previous studies [39].

### 4.2. Breeding Population of Maize Hybrids

The second population (Pop2) is a breeding population consisting of 523 hybrids derived from 246 inbred lines belonging to five different heterotic groups, based on a sparse diallel cross design [40]. The maize materials were cultivated in Yangzhou, Jiangsu Province, and Tai’an, Shandong Province, during the year 2017 and 2018. The experimental design employed was a randomized block design with two replications. Each experimental plot had two rows with a length of 3 m and a width of 0.5 m, with 13 plants planted in each row. For each hybrid, five uniform plants were selected to assess three traits: EW, EGW, and PH. The BLUP values for all traits across the two environments were computed for each hybrid using a mixed linear model implemented in the lme4 package in R 4.3.3 [41].

### 4.3. Genotype Data of Training and Breeding Populations

In the training population, genotype data for 5.820 hybrids were inferred from the genotypes of their respective parent inbred lines. Whole-genome resequencing was conducted on all 1.428 maternal lines and 30 paternal testers, resulting in the identification of 14.8 million single nucleotide polymorphisms (SNPs) [42]. Within the breeding population, 246 inbred lines were genotyped using genotyping by sequencing (GBS), yielding a total of 415.088 SNPs [40]. We screened the genotypes of the 224 inbred lines involved in the 5.820 hybrids, identifying 93.189 SNPs that overlapped with those of the breeding population. Missing genotypes in the hybrids due to heterozygosity of the parents were imputed using Beagle V4.0 [43]. SNPs were filtered with minor allele frequency (MAF) less than 0.05. Ultimately, 58.876 SNPs common to both populations were retained for subsequent analysis.

### 4.4. Training Population Optimization Methods

The kinship matrix was calculated using the kin function in R/predhy package. Note that the *K* matrix is partitioned into 2 × 2 blocks as below:

(1) $$\left[\begin{array}{cc} K_{G11} & K_{G12}\\ K_{G21} & K_{G22} \end{array}\right]$$

where $K_{G11}$ and $K_{G22}$ represent the kinship matrices for the training population and breeding population, respectively. The off-diagonal elements $K_{G12}$ and $K_{G21}$ represent the kinship between hybrids in the breeding population and those in the training population. We calculated the mean relationship criterion (MeanRel) as described by Brault et al. [24], which represents the average additive relationship between each genotype within the training population and all genotypes in the breeding population.

### 4.5. The PSAPGP Strategy

The PSAPGP strategy integrates population structure as a fixed effect in across-prediction models. Principal component analysis (PCA), clustering, and Q-matrix analysis were used to assess the population structure of 6.343 maize hybrids from training and breeding populations. The PCA was performed using the R/prcomp function, which enabled the identification of stratified clustering patterns and provided a summary of the genetic variation present across all maize hybrid genotypes. For clustering, we applied the Partitioning Around Medoids (PAM) method, implemented through the R/cluster package. PAM effectively categorizes objects into distinct clusters by minimizing the total dissimilarity between the objects assigned to a cluster and a central representative object, known as the medoid. This method is particularly beneficial for designing training sets, as it not only partitions all objects into clusters but also identifies representative objects for each cluster. The population structure Q-matrix was estimated using ADMIXTURE software, which employs a maximum-likelihood approach to estimate individual ancestries from multi-locus SNP genotype datasets.

The GBLUP and BayesB models were used to demonstrate the effectiveness of PSAPGP. The GBLUP model for PSAPGP is described as:

(2) $$y=Q\beta +Z\gamma_{G}+\varepsilon$$

where *y* is the observed vector of the hybrid phenotypic values; *Q* is an n×l design matrix for the fixed effect *β*; *Z* is an n×g design matrix for the additive effect $\gamma_{G}$; and *ε* is a vector of residual errors with an assumed $N\left(0,I_{n}\sigma^{2}\right)$ distribution. Assume that $\gamma_{G}\sim N\left(0,I_{g}\phi_{G}^{2}/g\right)$, where $\phi_{G}^{2}$ is the polygenic variance of additive effect. The parameters $\left\{\beta ,\;\phi_{G}^{2},\;\sigma^{2}\right\}$ were estimated using the restricted maximum likelihood method described in our previous study. Then, the parameters estimated from the training population were used to predict the phenotypic values of the breeding population.

The BayesB model for PSAPGP is described as:

(3) $$y=1n\mu +Q\beta +Z\gamma_{G}+\varepsilon$$

where *µ* is an intercept; *Q* is a design matrix for the effects of population structure; and *β* is the corresponding vector of effects, which were treated as ‘fixed’. *Z* is the genotype matrix and $\gamma_{G}$ the corresponding vector of marker effects which were treated as random. BayesB assumes that the prior distribution of the variances across markers is a two-component mixture of a point of mass at zero and a scaled-t slab [2]. BayesB were performed using R/BGLR package with all parameters set to default values [44].

## 5. Conclusions

In this study, we utilized a public dataset of maize hybrid as a training population to facilitate across-population genomic predictions for breeding populations. The optimization of the training population based on kinship yielded varying levels of improvement across different traits, with a more obvious advantage for traits with low heritability. We proposed a PSAPGP strategy, which integrates population structure as a fixed effect in the GS models. PCA, clustering, and Q-matrix analysis were used to assess the population structure. Notably, when the Q-matrix was used, the across-population prediction exhibited the best performance. This marks the first application of a public maize dataset for conducting across-population genomic predictions in maize hybrids. Improving the accuracy of across-population genomic prediction based on training population optimization and the PSAPGP strategy will play a key role in future maize hybrid breeding.

## Data Availability

The original contributions presented in the study are included in the article, further inquiries can be directed to the corresponding author.
